# Oxidative Stress and Inflammation in Hepatic Diseases: Therapeutic Possibilities of *N*-Acetylcysteine

**DOI:** 10.3390/ijms161226225

**Published:** 2015-12-18

**Authors:** Kívia Queiroz de Andrade, Fabiana Andréa Moura, John Marques dos Santos, Orlando Roberto Pimentel de Araújo, Juliana Célia de Farias Santos, Marília Oliveira Fonseca Goulart

**Affiliations:** 1 Pós Graduação em Ciências da Saúde (PPGCS), Campus A. C. Simões, Tabuleiro dos Martins, 57072-970 Maceió, AL, Brazil; kiviaqueiroz@hotmail.com (K.Q.A.); fabianamoura_al@hotmail.com (F.A.M.); 2 Faculdade de Nutrição/Universidade Federal de Alagoas (FANUT/UFAL), Campus A. C. Simões, Tabuleiro dos Martins, 57072-970 Maceió, AL, Brazil; jcfs_nut@yahoo.com.br; 3 Instituto de Química e Biotecnologia (IQB), Universidade Federal de Alagoas (UFAL), Campus A. C. Simões, Tabuleiro dos Martins, 57072-970 Maceió, AL, Brazil; john-sk8@hotmail.com (J.M.S.); orlando_rpa@hotmail.com (O.R.P.A.)

**Keywords:** *N*-acetylcysteine, liver, hepatic injury, antioxidant, anti-inflammatory, biomarkers

## Abstract

Liver disease is highly prevalent in the world. Oxidative stress (OS) and inflammation are the most important pathogenetic events in liver diseases, regardless the different etiology and natural course. *N*-acetyl-l-cysteine (the active form) (NAC) is being studied in diseases characterized by increased OS or decreased glutathione (GSH) level. NAC acts mainly on the supply of cysteine for GSH synthesis. The objective of this review is to examine experimental and clinical studies that evaluate the antioxidant and anti-inflammatory roles of NAC in attenuating markers of inflammation and OS in hepatic damage. The results related to the supplementation of NAC in any form of administration and type of study are satisfactory in 85.5% (*n* = 59) of the cases evaluated (*n* = 69, 100%). Within this percentage, the dosage of NAC utilized in studies *in vivo* varied from 0.204 up to 2 g/kg/day. A standard experimental design of protection and treatment as well as the choice of the route of administration, with a broader evaluation of OS and inflammation markers in the serum or other biological matrixes, in animal models, are necessary. Clinical studies are urgently required, to have a clear view, so that, the professionals can be sure about the effectiveness and safety of NAC prescription.

## 1. Introduction

The liver has multiple functions and is the principal detoxifying organ, acting in the clearance of pathogens, toxic chemicals and metabolic waste products from the body, also contributing, for the adequate function of other organs. It impacts heavily almost all physiologic systems to maintain homeostasis [1,2,3]. The continuous exposure of the liver to some factors, such as viruses, alcohol, fat, biotransformed metabolites, among others, can cause hepatic injury, which can lead to inflammation and liver degeneration. When the injury is sustained for long time, it can cause chronic liver diseases (CLDs), which occur in multistage processes of fibrosis, cirrhosis and hepatocellular carcinoma (HCC) [1]. Liver disease has a high prevalence in the world. For some patients, complications may occur, including portopulmonary hypertension, hepatorenal and hepatopulmonary syndromes [4].

Liver fibrosis is a wound healing process, which is reversible and results from chronic liver injuries, including those caused by alcohol consumption, chronic viral hepatitis, autoimmune diseases, parasites, metabolic diseases, lipopolysaccharide (LPS) [5] and other toxins or drugs [6,7]. In these cases, generally, the inflammation is the first phase, and develops to fibrosis after chronic oxidative stress (OS) [8]. When fibrosis is not controlled, it can progress into cirrhosis and after to HCC [7,9]. Alcoholic liver disease (ALD), nonalcoholic fatty liver disease (NAFLD) and chronic viral hepatitis (B and C), are the three most common causes of liver cirrhosis [7].

With the progression to chronic stage, there is activation of the immune system innate and adaptive, with polymorphonuclear leukocytes (PMN) infiltration, inducible nitric oxide synthase (iNOS) upregulation, and recruitment of lymphocytes through the portal tract, hepatic vein and sinusoids. Leukocytes and Kupffer cells (KCs) produce large amounts of nitric oxide (NO^·^) and cytokines, particularly transforming growth factor β (TGF-β) and tumor necrosis factor α (TNF-α), potent profibrogenic cytokine and inflammation modulator, respectively [10].

Reactive oxygen species (ROS) released by KCs activate the hepatic stellate cells (HSCs) leading to an increase of proliferation and synthesis of extracellular matrix (ECM), contributing to fibrosis and cirrhosis [8]. OS associated with inflammation causes focal or zonal necrosis, destruction of hepatocytes and architectural disarray [1].

**Figure 1:**
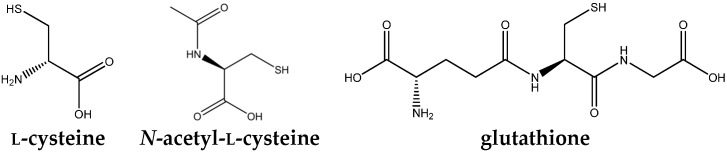
Chemical structures of *N*-acetyl-l-cysteine, l-cysteine and glutathione.

Antioxidants are natural or synthetic compounds, produced *in vivo*, normal cell constituents, or delivered in diets, whose main function is to fight against oxidative stress [11], being thus able to either delay or prevent the oxidation of substrates, such as proteins, deoxyribonucleic acid (DNA), lipids, lowering OS, DNA mutations, malignant transformations, as well as other parameters of cell damage [12]. Due to the role of hepatocytes in the metabolism of drugs, xenobiotics and endogenous compounds, these cells become the target of reactive oxygen and nitrogen species (ROS/RNS), giving reactive oxygenated metabolites (ROM), requiring, therefore, an important antioxidant defense system [13]. Among these antioxidant systems, stands out glutathione (GSH) (Figure 1), the most abundant cellular thiol antioxidant, which exhibits numerous and versatile functions and therefore protects cells against toxicity [14].

Several studies about hepatic diseases indicate that an overproduction of ROS/RNS and/or reduction of hepatic GSH are common profiles in these diseases, regardless their etiology [15].

*N*-acetyl-l-cysteine (NAC) (Figure 1) is known as an antioxidant that acts directly and/or by increasing intracellular GSH, especially on hepatic tissue [16]. NAC has an optimal thiol redox state, which is of great importance to optimize the protective ability of the cell to counter balance OS and inflammation [17]. Administration of NAC has been reported to be beneficial in other chronic clinical conditions, such as inflammatory bowel disease [18] obstructive pulmonary disease and other pulmonary diseases, systemic sclerosis, cystic fibrosis, human immunodeficiency virus (HIV) infection, septic shock, diabetes along with hepatic injuries [19].

Therefore, as the liver is a susceptible organ to oxidative damage and inflammation and considering the importance of NAC as an antioxidant in various conditions where OS and inflammation are involved, the objective of this review was to examine experimental and clinical studies that evaluated the antioxidant and anti-inflammatory roles of NAC, in attenuating respective markers of inflammation and OS in hepatic damage. Several positive results have been reported for the use of NAC, mainly concerning animal models. However, a standard experimental design of protection and treatment and the increase of human studies are necessary so that the professionals can be sure about the effectiveness and safety of NAC prescription.

## 2. Methods

We carried out a systematic review on articles published in PubMed, CAPES Periodicals (http://www.periodicos.capes.gov.br/) and Science Direct (http://www.sciencedirect.com/), using the following keywords: *N*-acetylcysteine/*N*-acetyl cysteine; liver; hepatic damage or injury liver, grouped in several ways to optimize the search. References cited in publications that were found were also used. Articles in which NAC was used alone through oral supplementation/administration by gavage, in diets or liquids; via intramuscular (i.m.); intraperitoneal (i.p.); subcutaneous (s.c.) and intravascular (i.v.), in studies *in vivo* (rats or mice—in order to minimize differences between species), *in vitro* (rats, mice and human hepatocytes) and in humans that evaluated the protective role and/or treatment in liver injury by attenuation of OS and inflammation markers in tissue and plasma samples, were chosen. To minimize losses, the search was conducted by two researchers, independently.

Initially, the papers titles were read for initial selection and exclusion of duplicated articles. Thereafter, the abstracts were read, and subsequently, the articles that did not fit the inclusion criteria or did not make clear the model of pre-treatment or treatment using NAC and its form and route of administration, were excluded. The articles were grouped into the following categories: diseases, drugs, alcohol, pesticides, ionizing radiation and others.

## 3. Results

Sixty-nine articles were found, among them, only 1 (1.4%) article was related to test conducted in humans and 54 (78.2%) articles related to *in vivo* tests and 14 (20.1%) articles to *in vitro* ones. In common, these articles had shown oxidative stress and inflammation involved in the pathogenesis of liver damage. The results related to the supplementation of NAC with antioxidant and anti-inflammatory roles in any form of administration and type of study are satisfactory in 85.5% (*n =* 59), considering some markers of oxidative stress and inflammation (Table 1), in cases evaluated as liver damage.

**Table 1:** Markers of antioxidant defense, oxidative damage and inflammation.

Synthesis of RONS or Damage by Them, Forming ROMs	Increase of AO Defense	Cytokines and Interleukins Synthesis and Levels
Levels of: O_2_·^−^, H_2_O_2_, HO·, HOCl, NO·, ONOO^−^, ONOOH, NO_2_·^−^, ROS; NO(x)	Antioxidant enzymes: GPx, GR, GST, SOD, CAT	Levels of: TNF-α, IL-1β, INF-γ, IL-6, IL-10, IL-1, IL-5, IL-12, IL-16, IL-2, IL-4, IL-17, TGF-β
Levels of enzymes (activity/expression) related to RONS: iNOS, XO, COX, LPO, MPO; cytochrome P450 2E1, PGE_2_	Levels of non-enzymatic defenses: GSH, GSH/GSSG	
Levels of producing enzymes (activity/expression) related to pro-oxidants: NF-kB, IkB-α	P-SH, Vit E, Vit C, Zn, Se; Levels of antioxidant capacity (TAS, TAC)	
Levels of oxidative damage caused by RONS: formation of AGE, 8-OXOdG, LP, MDA, 4-HNE, TBARS, GSSG, protein carbonyls	Levels of producing enzymes (activity/expression) of antioxidants: Nrf2	

AO: antioxidants; AGE: advanced glycation end-products; CAT: catalase; COX: cyclooxygenase; GPx: glutathione peroxidase; GSH: reduced glutathione; GSSG: oxidized glutathione; GSH/GSSG: ratio reduced glutathione/oxidized glutathione; GR: glutathione reductase; GST: glutathione *S*-transferase; IL: interleukin; iNOS: inducible nitric oxide synthase; INF-γ: Interferon gamma; IkB-α: nuclear factor of kappa light polypeptide gene enhancer in B-cells inhibitor, α; LPO: lipoxygenase; LP: lipid peroxide; MPO: Myeloperoxidase; MDA: malondialdehyde; NO(x): total nitrate/nitrite; NF-kB: nuclear factor kappa-light-chain-enhancer of activated B cells; Nrf2: Nuclear factor (erythroid-derived 2)-like 2; PGE_2_: prostaglandin E2; P-SH: thiolated protein; ROMs: reactive oxygen metabolites; ROS: reactive oxygen species; RONS: reactive oxygen and nitrogen species; SOD: superoxide dismutase; TAC: antioxidant capacity; TAS: total antioxidant status; TGF-β: transforming growth factor β; TBARS: thiobarbituric acid-reactive substances; XO: xanthine oxidase; 4-HNE: 4-hydroxy-2-nonenal; 8-OHdG: 8-oxo-7,8-dihydro-2 deoxyguanosine.

## 4. Discussion

### 4.1. Oxidative Stress and Inflammation in Hepatic Lesion

The liver is the main organ in the metabolism and detoxification of substances derived from the ingesta and also produced from blood. It is equipped with a number of resident innate immune cell populations including KCs, dendritic, natural killer (NK), and natural killer T (NKT) cells, all contributing to numerous liver pathologies [20]. Under normal circumstances, the liver aerobic metabolism leads to the production, in a dynamic steady state, of pro-oxidants, such as ROS and reactive nitrogen species (RNS), which are balanced by their sequestration by antioxidants, with a similar rate [21]. At physiological levels, ROS have an important biological role, and are considered vital cellular mediators in various metabolic and signaling pathways. These functions, although well documented, are still controversial [22,23].

OS, an imbalance between oxidants and antioxidants in favor of the oxidants, is involved in the pathogenesis of liver diseases [24], regardless their etiology and disease course. A common basis in all types of hepatic injury is the increase in ROS generation [25], and, for that, OS biomarkers may be helpful as indicators of pathogenic processes, or pharmacological responses to therapeutic intervention, and may be used in diagnosis, prognosis and individualized therapy [26].

The term ROS is generic and describes a wide variety of molecules, free radicals (chemical species with unpaired electrons) or ions derived from molecular oxygen, for instance, singlet oxygen (O_2_), superoxide anion radical (O_2_·^−^), hydrogen peroxide (H_2_O_2_) and hydroxyl radical (HO·) [27], among others. Main sources of ROS are the mitochondria and the cytochrome P450 in the hepatocyte, KCs and neutrophils [25]. Such species have different chemical reactivity, half-life and lipid solubility. The toxicity and high reactivity of ROS result from their short half-life, which in turn limits their diffusion. Contrary to ROS, the aldehydic products such as malondialdehyde (MDA) and 4-hydroxynonenal (4-HNE) have longer half-lives and, thus, are able to diffuse from their site of origin to other places (intra and extracellular), amplifying the effects of OS [28]. These products are generated by lipid peroxidation in cell membranes and organelles due to damage by ROS in polyunsaturated fatty acids (PUFAs) [29,30,31].

ROS and RNS may be removed by the liver by antioxidants, such as GSH, vitamins C, A and E, and enzymes such as superoxide dismutase (SOD), catalase (CAT), glutathione peroxidase (GPx) and thioredoxin [32,33]. SOD is involved in the dismutation of O_2_·^−^ to H_2_O_2_, and CAT or GPx transforms H_2_O_2_ to water and free oxygen. The hydroxyl radical, HO·, is formed by Fenton and/or Haber-Weiss reactions, with transition metals as catalysts [34].

OS may be observed in several etiologies of liver diseases such as NAFLD, ALD, drugs and pesticides intoxication, ionizing radiation, toxins and other chemicals [14]. Moreover, the liver may be injured during the course of many diseases that are systemic or predominantly involve other organs, as sepsis, cardiovascular diseases (CVD), obesity, chronic kidney disease (CKD) and diabetes [35]. Despite the large number and variety of initiating factors, derived from extra- or intrahepatic environments [25,35,36], the final results are restricted, including death of hepatocytes or cholangiocytes leading, in severe cases, to the stereotyped anatomical and clinical patterns of portal hypertension, with or without cirrhosis [35].

A common source of OS in liver diseases emanates from activated hepatic phagocytes, the KCs [25], also known as Browicz-Kupffer cells and stellate macrophages [37], one of the resident innate immune cell populations [20]. They participate in all forms of chronic inflammatory liver diseases [25] and their involvement in these diseases can represent their recruitment as a tissue response to OS [25,37,38].

Activated KCs, through a nuclear factor κ-light-chain enhancer of activated B cells (NF-κB) mediated mechanism, produce a complex and highly interactive repertoire of inflammatory mediators and cytokines [25], such as TNF-α, interleukins IL-1β, IL-6, IL-12, IL-18 and iNOS [39], as well as activate them to produce oxidants including superoxide derived nicotinamide adenine dinucleotide phosphate-oxidase (NADPH) and endocytose bacteria carried through the portal circulation [20]. NF-κB represents a family of proteins that share the DNA binding domain known as the Rel homology domain (RHD) in the form of homo- or hetero-dimers (p50/p65) and activate many genes involved in cellular response to OS, including genes to cytokines, growth factors, adhesion molecules, and acute phase proteins [40]. The NF-κB dimers are non-covalently bound to an inhibitory protein IκB that prevents its nuclear translocation. After their phosphorylation by specific IκB kinases (IKK), and poly-ubiquitination, proteolytic degradation occurs by the 26S proteasome with subsequent transport of NF-κB to the nucleus [41].

NADPH may stimulate, in hepatocytes, the production of ROS [42] that cause DNA damage, induce apoptosis, the expression of genes involved in the synthesis of pro-inflammatory cytokines, triggering, in the worst outcome, their transformation into malignant cells. iNOS may stimulate hepatocyte toxicity by increasing the production of NO· [43]. Some of these cytokines may activate specific intracellular pathways, *i.e.*, pro-apoptotic signals via caspase cascade [25,44]. In some liver disorders, apoptosis eliminates a critical number of hepatocytes, leading to impaired liver function [25]. Injured hepatocytes may release apoptotic bodies and activate KCs, and these activated cells can promote inflammatory and fibrogenic responses, leading to a vicious cycle of hepatic injury [43].

During inflammation, the cells of the immune system, among which mast cells and leukocytes, are recruited to the place of the injury, which leads to an exacerbation of cellular respiration due to increased oxygen consumption causing an increase in release and accumulation of ROS at the site of damage [45].

The activation of KCs amplifies tissue inflammatory responses also through attraction and adhesion of neutrophils and mast cells, and by release of compounds that provoke the clumping together of platelets, obstructing local microcirculation, leading to ischemia reperfusion [25]. The generation of these inflammatory mediators, ROS, overexpression of pro-apoptotic proteins, depletion of antioxidants and mitochondria damage is associated with morphological and functional changes that induce an acute inflammatory response leading to several clinical complications [43,44]. Examples of this are: fibrosis, defined as the accumulation of excessive ECM; and later cirrhosis, characterized by the loss of normal liver architecture and formation of septae and nodules, portal hypertension and progress to hepatocellular carcinoma and hepatic insufficiency [10,46].

The cytokines and chemokines derived from KCs, such as TGF-β1, platelet-derived growth factor (PDGF), TNF-α, and IL-1, as well as the damage to hepatocytes, can induce the activated hepatic HSCs, formerly known as fat-storing cells, Ito cells, lipocytes, perisinusoidal cells, or vitamin A-rich cells [37], to transform into myofibroblasts, promoting hepatic fibrogenesis [7,10,43]. The progressive activation of HSCs leads to changes in cellular functions, generating subpopulations of stellate cells with different phenotypic profiles. Moreover, the activation of HSCs is associated with increased smooth muscle α-actin expression (αSMA) [7,47] and production of ECM components, such as collagen types 1, 3 and 4 [37,39,48]. Mediators released after hepatocytes’ damage, such as MDA/4-HNE, cytokines, ROS/RNS and hepatotoxins are important activators of HSC [10]. With the persistence of these stimuli, and maintenance of injuries, a perpetuation phase regulated by autocrine and paracrine stimulation, is created. This phase promotes changes in HSC behavior, including proliferation, contractility and fibrogenesis [49].

ROS also cause the crosslinking of cytokeratins to form Mallory corpuscles and stimulate neutrophil chemotaxis [28]. Besides that, these reactive species promote the up-regulation of the expression of genes involved in fibrogenesis, such as pro-collagen type I, monocyte chemoattractant protein 1 (MCP-1), and tissue inhibitor of metalloproteinase-1 (TIMP1), possibly via activation of transcription factors, including c-jun N-terminal kinases (JNKs), activator protein 1 (AP-1), and NF-κB [10]. MDA also contributes to inflammation through the activation of NF-κB and 4-HNE, which are profibrotic stimulus, up-regulating procollagen and tissue inhibitor of *TIMP1* gene expression [8].

The activation of HSCs stimulates the release of pro-inflammatory cytokines, causing inflammation which initiates fibrogenesis, apoptosis and hepatocyte necrosis [39]. Moreover, this hepatic inflammation favors the recruitment of immune and inflammatory cells. When the hepatic stellate cells are deactivated, this facilitates the completion of fibrogenesis and regression of extracellular matrix [25,43].

In this context, all these molecular and cellular interactions and their perpetuation, might lead to fibrosis, cirrhosis and hepatocellular carcinoma [43].

### 4.2. N-Acetylcysteine and Its Antioxidant and Anti-Inflammatory Properties

Many current studies about treatment of hepatic diseases are contradictory and not enough clear. However, an indisputable area of therapeutic benefit involves attenuation of oxidative liver damage. As reported, the mechanisms of hepatic injury are multi-factorial, but almost all involve oxidative injury in cell membrane and organelles, proteins, enzyme and lipids, and the treatment, in part, involves protection against oxidative injury and approaches able to maintain an appropriate redox balance [25]. Antioxidants, natural or synthetic, have been of special use for this control and some of them must prolong survival and reduce occurrence of HCC [43]. Thus, antioxidant activity plays a key role in alternative therapeutics, involving the liver [25].

Thiols, the most important systemic and intracellular endogenous antioxidants, have been the focus of particular interest of scientific community [25,50,51]. Thiols can be found either in proteins (thiolated proteins, P-SH) or in low molecular mass metabolites, including GSH (Figure 1) [32]. Altered thiol status has effects on hepatocytes and other resident and transient cells within the liver [25]. The increase of the thiol levels of the cell, by the direct administration of GSH, is not recommended, once GSH does not easily cross the cell. It is synthesized within the cell [52]. Therefore, alternatives are necessary for inducing the synthesis of glutathione, as the supply of precursor amino acids and/or other inducing agents [53]. As a source of -SH groups, *N*-acetyl-l-cysteine (NAC) (the active form), is being studied in diseases characterized by increased OS or decreased GSH [54]. Low antioxidant capacity in a cellular environment with OS is mainly due to the decrease in GSH and/or increase of oxidized glutathione (GSSG), once GSH is the most abundant intracellular thiol, present in millimolar concentrations in many cell types, including the liver [32,50,51]. GSH is directly involved in the removal of toxic aldehydes and peroxides and indirectly maintains the reduced state (active form) of vitamins C and E [55]. Moreover, GSH acts in the detoxification of electrophilic xenobiotic compounds, modulation of the redox regulation in signal transduction, of the of immune response, metabolism of prostaglandins and leukotrienes, of neurotransmitter signaling, of modulation of cell proliferation, among others [40]. Therefore, the NAC could confer benefits in disorders caused by the OS [56]. It was first reported to have clinical benefits in the early 60^ties^, when it was observed that NAC had a mucolytic agent effect in patients with cystic fibrosis [52]. However, conclusive evidence of its efficacy is still lacking [57].

Being a thiol (R-SH), NAC may be oxidized by various radicals and serves as a nucleophile [40]. Compared with cysteine (Cys), NAC has lower toxicity and susceptibility to oxidation (and dimerization), in addition to having enhanced solubility in water [50]. The aminoacid Cys is easily oxidized, generating the inactive disulfide, and relatively unstable cystine (Cys–Cys). It was originally believed that NAC might have the capability to entering in cell via membranes without aminoacid transporters, due to the reduced charge imparted by the acetyl moiety. In addition, some of its forms are not well absorbed, and transport activity of cells and tissues are low [52].

NAC is a reducing agent stronger than cysteine and GSH, showing a more negative redox potential in 63 and 106 mV *vs.* Orion 9103BN semi-micro combination electrode, than the GSH/GSSG redox couple and Cys/Cys-Cys, respectively [58]. As for the direct elimination capacity of reactive oxygen and nitrogen species (RONS) [59], NAC reacts quickly with highly oxidant radicals, at pH 7 and at room temperature, for instance HO· (1.36 × 10^10^ M^−1^·s^−1^), nitrogen dioxide (NO_2_·) (≈1.0 × 10^7^ M^−1^·s^−1^) and carbonate radical (CO_3_·^−^) (≈1.0 × 10^7^ M^−1^·s^−1^) [40]. Thus, NAC acts in the detoxification of ROS produced by leukocytes [60] and is able to chelate transition metals such as Cu^2+^, Fe^3+^ and heavy metals such as Cd^2+^, Hg^2+^ and Pb^2+^ [61], primarily through its thiol side chain to form complexes, which are easily excreted from the body, being removed from the intra- and extracellular media [40]. At a physiological pH, NAC may chelate Hg^2+^ and thus act as an antidote against HgCl_2_ poisoning [62]. Although NAC has the ability to directly scavenge free radicals, the constant reaction rate with ROS are smaller than in relation to the antioxidant enzymes such as SOD, CAT and GPx [63]. Thus, the direct elimination of radicals is not as important as its antioxidant activity.

There are various ways to provide this antioxidant: orally, i.v., topically or by inhalation. The administration of NAC orally is preferred, despite some clinical situations that require i.v. administration [64]. Oral NAC is usually well accepted and its indication as “nutraceutical” has a positive impact on patients [65]. In Europe, NAC is produced and packed in capsule and tablet forms, as well as in effervescent formulations, which can be dissolved in water and juices to create a pleasant flavor, being easily tolerated and absorbed [50], and in eye drops [66]. There are not recommendations for oral dosing with NAC and consequently a broad range of doses have been used in clinical trials. Mild nausea, vomiting and diarrhea have been reported as dose-dependent side effects of oral NAC [50,65].

NAC consumed orally is absorbed in the stomach and intestine and is delivered to the liver via the portal vein. In the liver, NAC, quickly, integrates peptides for the generation of proteins and a diversity of metabolites [58,67,68]. In plasma, NAC may be present in its form reduced and in various oxidized forms. It may be oxidized to a disulphide, the *N*,*N*′-diacetylcystine, and it may react with other low molecular mass thiols, such as Cys and GSH, forming mixed disulfides. In addition, NAC can suffer redox reactions with thiol groups of the plasma proteins and become oxidized [65].

**Figure 2:**
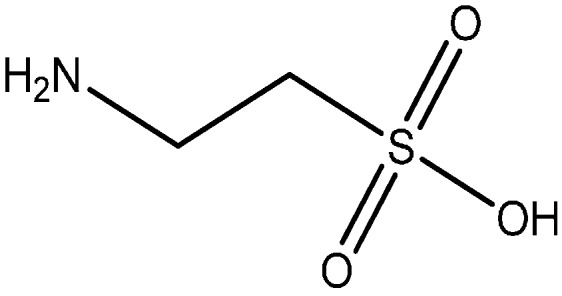
Chemical structure of taurine.

NAC is almost completely absorbed, when orally administered to rats, only 3% of the radioactivity of ^35^S-NAC being excreted in the faeces [69], indicating that the absorption of NAC and its metabolites is almost complete [68]. A metabolic study with ^35^S-NAC in rats showed that Cys and Cys-Cys were the major metabolites in the liver and inorganic sulphate was the major urinary excretion product [70]. Between 13% and 38% of an oral dose of radioactive NAC are detected in the urine after 24 h [71]. In addition, small amounts of taurine (Figure 2) and sulphates are also detected in the urine [68].

**Figure 3:**
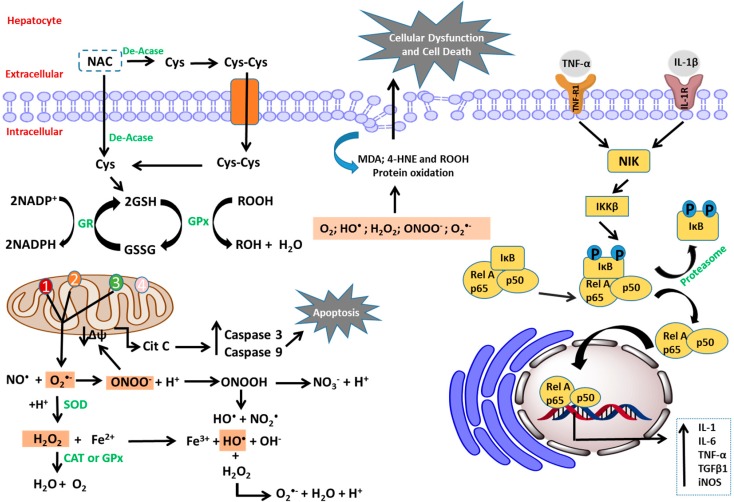
Transport of *N*-acetyl-l-cysteine and pathways for the generation of oxidative stress and inflammation in hepatocyte. Legend: CAT: catalase; Cys: cysteine; Cys-Cys: cystine; Cit c: cytochrome c; De-Acase: deacetylases; GSH: reduced glutathione; GSSG: oxidized glutathione; GPx: glutathione peroxidase; GR: glutathione reductase; HNE: 4-hydroxynonenal; iNOS: inducible nitric oxide synthase; IKKβ: inhibitor of κB kinase; IκB: inhibitor of NF-κB; IL: interleukin; IL-1R: interleukin-1 receptor; MDA: malondialdehyde; NADP^+^: oxidized nicotinamide adenine dinucleotide phosphate; NADPH: reduced nicotinamide adenine dinucleotide phosphate; NIK: NF-κB-inducing kinase; NO·: nitric oxide; NAC: *N*-acetylcysteine; NF-κB: nuclear factor κ-light-chain enhancer of activated B cells; p65: nuclear factor NF-κB protein p65 subunit; p50: nuclear factor NF-κB protein p50 subunit; Rel A: v-rel avian reticuloendotheliosis viral oncogene homolog A; ROOH: organic hydroperoxide; ROH: alcohol; SOD: superoxide dismutase; TGFβ1: transforming growth factor β 1; TNF-α: tumor necrosis factor α; TNF-R1: TNF-α receptor 1; ΔΨ: mitochondrial membrane potential; 1: NADH-ubiquinone reductase; 2: succinate-ubiquinone reductase; 3: ubiquinol-cytochrome c reductase; 4: cytochrome c oxidase.

By extracellular deacetylation of NAC, Cys is released and introduced into cells via amino acid transporters [53]. The process of deacetylation of NAC remains unclear. The hypothesis is that the free Cys is required for the synthesis of GSH. NAC crosses the intact cell membrane before suffering hydrolysis to cysteine within the cell with the action of *N*-deacetylases. For mucolytic effects of NAC, deacetylation may not be a precondition, once the sulfhydryl group is free to interact with the disulfide bonds of the protein. It is not affected by the presence of the acetyl group (Figure 3) [52]. Oral bioavailability is considered to be low (<10%), potentially due the intestinal absorption and high first-pass metabolism. However, in plasma, the level of NAC is low, once it is rapidly introduced into cells and converted to GSH, keeping a constant concentration gradient through the membrane [71].

The oral bioavailability of NAC in humans, calculated as the area under plasma concentration-time curve (AUC) (oral)/AUC (i.v.), varied between 6% and 10%, probably due the intestinal absorption and high first-pass metabolism [71]. The plasma levels of NAC, after oral administration of 600 mg, reach its maximum after 60 min (4.6 μM), rapidly decreasing to 2.5 μM, after 90 min. Other studies have described values of 16 and 35 μM, following oral administration of 600 to 1200 mg/day, respectively. The plasma half-life is suggested to be about 2.5 h and NAC is not detecTable 10–12 h after administration. After of an intravenous administration, high concentrations of NAC in the plasma are observed. To inhibit inflammation in *in vitro* studies, the NAC concentration should be higher than that required to inhibit OS. Already in *in vivo* studies, higher doses are necessary to provoke acute antioxidant and anti-inflammatory effects. But to perform *in vivo* chronic studies, lower doses are required for the same effect [59].

As with any drug, NAC has side effects and adverse reactions. Due to its high osmolality, a high therapeutic dose of NAC can lead to confusion and electrolyte disturbances [56]. Adverse actions are not clinically recognized with the oral administration of NAC at doses up to 8000 mg/day [72]. It is apparent that high levels of antioxidants may have a pro-oxidant effect, possibly mediated by the reduction of transition metals and enhancement of Fenton Chemistry [52].

The results of toxicological studies with NAC were reviewed by Bonanomi and Gazzaniga (1980) [52]. According to this review, acute, sub-acute and sub-chronic toxicity studies were performed in rats and dogs, teratologic and reproduction studies in rats and rabbits and a mutagenicity test *in vitro*. In acute toxicity, the oral LD_50_ in mice and rats was determined to be greater than 6000 mg/kg body weight (b.w.). In the studies that observed the sub-acute and sub-chronic toxicity, oral administration from 2000 mg/kg b.w./day over 4 weeks in male and female Sprague-Dawley rats and 1000 mg/kg b.w./day over 28 weeks in male and female Sprague-Dawley rats did not affect behaviour, body weight gain, hematology, hepatic and renal function, prothrombin and bleeding time. Necropsy findings and histological examinations did not provide evidence of pathological lesions. An Ames test using *Salmonella typhimurium* with and without hepatic microsomes did not show a mutagenic effect [69].

Positive results were observed with the use of NAC in animal models [73] and in clinical trials [74] and has been used in the treatment of various disorders, such as intoxication [40], cardiac ischemia-reperfusion (I/R) injury [75], chronic obstructive pulmonary disease (COPD) [59], bronchitis [50], HIV [76], psychiatric disorders [77], and others.

In many studies, NAC was shown to increase GSH levels in hepatic cells, having, in turn, antioxidant effects in cells. It increased the protection capability of hepatic cell, since the exhaustion of GSH is often seen as a consequence of the increased OS and inflammation [78]. NAC stimulates also the activity of cytosolic enzymes involved in GSH request, such as GSH reductase (GR) [53].

Other beneficial effects of NAC are: (1) reduction of disulfide bonds of the protein, disrupting its links and changing their structures, which may explain its activity in TNF-α blocking by reducing the affinity for the receptor [79,80]; (2) regulation of cell cycle and apoptosis [81]; (3) anti-neoplastic and anti-mutagenic activity, including electrophilic metabolic blocking, either from endogenous or exogenous origin [82]; (4) modulation in gene expression and signal transduction; (5) modulation of the immune system and mitochondrial functions [40].

Regarding apoptosis, Lin *et al.* (1997) [83] demonstrated the protective effect of NAC against apoptosis induced by peroxynitrite (ONOO^−^) by modulating levels of O_2_·^−^ and H_2_O_2_. Zaragoza *et al.* (2000) [84] reported that NAC has been used to protect against cocain-induced apoptosis by upregulation of antioxidant enzymes such as SOD (Mn-SOD and Cu/Zn-SOD), GPx and CAT. In addition, NAC is well absorbed, it is valuable for immune cells, and influences various pathways of the phagocytic process [85]. NAC has shown immunomodulatory activity, improving immune functions such as chemotaxis of leukocytes and reducing levels of TNF-α and IL-8 [40], cytokines that attract inflammatory cells and increase oxidant production by these cells [86]. Yet, changing the thiol intracellular solubility, NAC may influence the balance of Th1 and Th2 cells, helping in the stimulation of specific cytokine profiles. Th1 cells principally secrete TNF-α and IL-12, both of them, pro-inflammatory cytokines, whereas Th2 cells primarily release IL-10, anti-inflammatory cytokine [85]. As certain immune properties are very sensitive to OS, even a moderate decrease in the intracellular GSH levels has important consequences for a variety of leukocyte functions, especially the more sensitive such as T lymphocytes proliferation and NK activity, dependent on IL-2 [53]. NAC inhibits the expression of vascular cell adhesion molecule 1 (VCAM-1), a member of the immunoglobulin superfamily, by inhibiting the binding of NF-κB to the VCAM-1 κB motif [87]. VCAM-1 regulates T-cell recruitment and allows that the inflammation persists, giving a survival signal for lymphocytes [88].

The anti-inflammatory activity of NAC has been studied through its effect on NF-κB, which is central in the regulation of expression of genes involved in the response to OS, inflammatory [41,59,85,89] and transcriptional pathways such as p38 and extracellular signal-regulated kinase (ERK1/2), stress-activated protein kinase/c-Jun kinase (SAPK/JNK), c-Jun and c-Fos, among others [59]. Along with these facts, it affects other transduction pathways, gene expression and modulates activity of transcription factors both *in vivo* and *in vitro* [40]. The treatment of cells in *in vitro* culture or in patients with sepsis using NAC suppresses NF-κB activation and subsequent cytokine production [90,91]. With the release of NF-κB, the transcription of genes encoding TNF-α, IL-1 and IL-6 can increase, which ultimately results in a cycle of positive feedback. TNF-α causes IκB ubiquitination and subsequent degradation by proteases. NAC blocks TNF-α activation of NF-κB regardless of their antioxidant activity, causing structural changes in the TNF-α receptor which reduces its receptor affinity α [80].

According to the study of Pajonk *et al.* (2002) [92], administration of NAC suppresses 19S catalytic subunit, thereby inhibiting the activation of NF-κB and according to Oka *et al.* (2000) [93], NAC inhibits IKK. NAC also inhibits the production of NO· by the inducible form of iNOS and IL-6 by cells of the immune system [80]. Pathophysiological effects are observed when NO· levels are elevated, due to its interaction with O_2_ or O_2_·^−^ [94]. Moreover, studies in rat livers and human cells have suggested that NO· has the capacity to induce apoptosis [95] and that caspase-8 gene expression induced by NO· could contribute to cell death [96].

Therefore, it is clear the potential of NAC as a therapeutic drug in various diseases in which OS and inflammation are involved in the pathogenic process.

**Figure 4:**
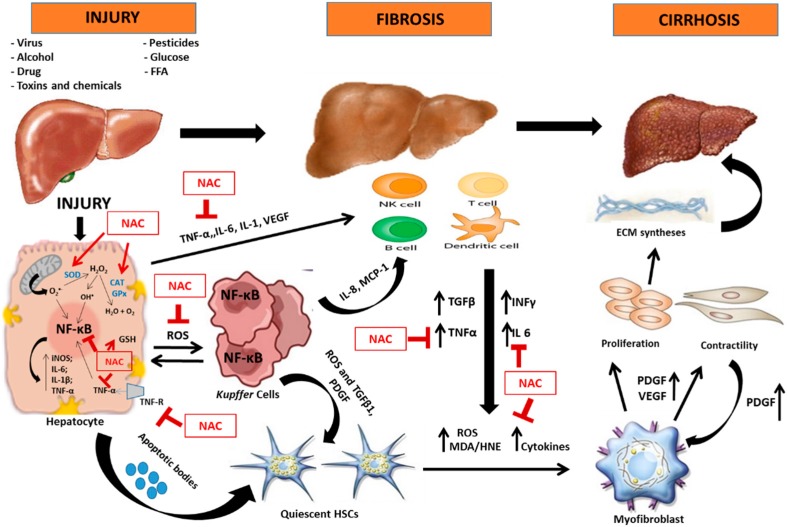
Possible molecular mechanisms of action of *N*-acetylcysteine (NAC) in attenuation of liver injury involving oxidative stress and inflammation. Adapted from Cohen-Naftaly; Scott L. Friedman, 2011. Legend: CAT: catalase; ECM: extracellular matrix; FFA: free fatty acids; GPx: glutathione peroxidase; HSCs: hepatic stellate cells; iNOS: inducible nitric oxide synthase; IL: interleukin; INFγ: interferon gamma; MCP-1: monocyte chemoattractant protein-1; MDA/HNE: malondialdehyde/4-hydroxynonenal; NF-κB: nuclear factor κappa-light-chain enhancer of activated B cells; PDGF: platelet-derived growth factor; ROS: reactive oxygen species; SOD: superoxide dismutase; TGFβ1: transforming growth factor β 1; TNF-α: tumor necrosis factor α; TNF-R: TNF-α receptor; VEGF: Vascular endothelial growth factor. 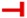 inhibition; 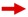 stimulation; ↑ increase.

In this section, we briefly describe the impact of OS and inflammation in liver diseases and the possibilities of use of NAC as a therapeutic agent (Figure 4).

### 4.3. Hepatic Diseases and Possibilities of Therapeutic Effect of N-Acetylcysteine

The mainly used markers of antioxidant defense, of oxidative damage and inflammation are represented in Table 1.

#### 4.3.1. Chronic Viral Hepatitis

##### Hepatitis C Virus

The major cause of chronic liver disease is hepatitis C virus (HCV). Epidemiological results, recently reported, show an increase in incidence over the last 15 years by 2.8%, reaching >185 million infections worldwide [97]. In such a situation, there is an increasing risk of evolution for cirrhosis and HCC. 23% of HCV patients develop HCC [98]. Despite the full understanding of the mechanisms by which HCV causes cellular damage, several evidences confirm that ROS production increases while antioxidant defense decreases significantly in all types of liver damage [99,100]. Therefore, OS are involved in the pathogenesis of chronic hepatitis C, as well as in the induction and progression of liver disease [101] and hepatocarcinogenesis in HCC [102].

One of the consequences of HCV gene expression is the endoplasmic reticulum stress (ER). This stress causes both decrease of ER calcium stocks and increase of calcium uptake in the mitochondria [103] and consequently stimulates ROS generation which is involved in alteration on the properties of proteins through lipid peroxidation, influencing, negatively, an important via in the host cell, including the oxidation of fatty acids and their export [104]. ROS activates cellular tyrosine and serine/threonine kinases, which then activate NF-κB and activator of transcription 3 (STAT-3). NF-κB is activated by the calcium/calpain-regulated degradation of IκBα and into the nucleus causes the activation of pro-inflammatory and pro-oxidant genes [105].

Another mechanism due to HCV infection appears to be closely involved with OS: the iron overload [106]. Some data in the literature show an obvious increase of Fe^+3^ contents both in the liver and in the serum of these patients [107,108], but the mechanism by which HCV regulates iron metabolism is poorly understood. As a consequence of hepatic iron accumulation, Fenton’s reaction and generation of HO· increase [34]. HO· reacts with lipid membranes, proteins and DNA, and causes liver damage observed by increase of serum alanine aminotransferase (ALT) [109].

Besides those aspects, redox imbalance in patients with HCV can also be caused by CAT gene polymorphism, which reduce endogen antioxidant defense [110]. Additionally, patients with HCV present vitamin A deficiency and this is correlated with unresponsiveness to interferon-based antiviral therapy [111]. It should be noted that vitamin A is considered an important exogenous antioxidant and more than 90% of total body vitamin A is stored in the liver. Moreover, ROS activate HSCs, which lead to hepatic fibrosis and the disease progression in patients with HCV [112]. Another redox alteration observed in patients with HCV, is the increase of MDA levels [113] and decrease of SOD and total antioxidant status (TAS) [114]. The inflammation, closely associated with OS, can be also identified in HCV progression, especially necrosis and apoptosis and persistence of this inflammation are associated with the progression of liver fibrosis and the development of cirrhosis [114].

Some nutrients with antioxidant and/or anti-inflammatory properties have been tested successfully in HCV treatment, for instance, vitamin E [115,116].

Until now, NAC has not been used in HCV treatment, its anti-fibrotic action in different animal models of liver disease can suggest positive effects, being necessary *in vivo* studies that evaluating the effect of NAC, but clinical trials are necessary to support its use.

##### Hepatitis B Virus

One of more prevalent public health problem worldwide is Hepatitis B virus (HBV) infection. This virus affect 30% of the world's population [117]. It is estimated that approximately 350–400 million people are chronically infected with HBV in the world and this infection can lead to liver fibrosis and progress to hepatocellular carcinoma [118]. Although the majority of patients with chronic HBV infection do not develop hepatic complications, it can cause serious illness later in life [119].

The HBV infection can alter the metabolism of host cells to sustain its replication and expression including the up-regulation of glutamate dehydrogenase 1 and isocitrate dehydrogenase [120], the transcriptional up-regulation of genes involved in lipid biosynthesis [121] and the promotion of both hexosamine and phosphatidylcholine biosynthesis [122]. Together with these metabolic alterations, OS also presents an important action on viral replication. Four open reading frames: denoted Cp (core protein), Sp (surface protein), Pol (polymerase), and HBx (X protein) are part of the double-stranded DNA genome of HBV and associated with OS, particularly with increase of H_2_O_2_ and decrease of GSH levels [123].

Metabolic changes caused by HBV are associated with fatty liver, inflammation and especially OS. In terms of OS, it is observed by GSH consumption [122], decrease of GPx activity and increase of MDA [124] and carbonyl [125] levels. Additionally, β-carotene [126], ceruloplasmin levels, total oxidant status (TOS), TAS, and OS index (OSI), represented by TOS (μmol H_2_O_2_ equivalent/L)/TAS (mmol Trolox equivalent/L), suggest that OS may be associated with hepatitis B activity [127]. Therefore, it had been demonstrated to have an effect on DNA damage and hepatocarcinogenesis [128].

Similarly to HCV, NAC has not yet been tested in HBV treatment. However, the facts already discussed can support its use in animal tests and further in clinical trials.

#### 4.3.2. Alcohol

Alcoholic liver disease (ALD) can manifest itself in different severity grades, going from hepatic steatosis (the simple form of liver damage) up to severe cirrhosis [129] ALD is the most common liver disease in world and its responsible for 3.8% of all deaths and 4.6% of disability-adjusted life-years in consequence of alcohol intake [130].

Classically there are three stages of ALD, which are histologically classified as: fatty liver or hepatic steatosis, alcoholic hepatitis, and chronic hepatitis with hepatic fibrosis or cirrhosis [131], and in all of them, inflammation and OS occur. The metabolization of alcohol to acetaldehyde, directly responsible for damage in liver, is promoted by key enzymes, including alcohol dehydrogenase (ADH), CYP2E1, and CAT [132]. Similarly to alcohol dehydrogenation to acetaldehyde by ADH, acetaldehyde dehydrogenation to acetate by its respective dehydrogenase (aldehyde dehydrogenase—ALDH), can lead to the synthesis of NADH, which inhibits fatty acid oxidation and promotes fat accumulation. NADH, in excess, is responsible for the indirect liver damage. Alternative metabolism of alcohol by CYP2E1 leads to the production of ROS, causing lipid peroxidation and inflammation. Furthermore, alcohol also increases intestinal permeability, leading to endotoxemia. This leads to the activation of KCs in the liver to release TNF-α, which in turn leads to more OS [133]. When acetaldehyde accumulates, it binds, forming adducts, *i.e.*, covalent chemical addition products, with proteins, lipids, and DNA, causing dysfunction and DNA damage and mutation [132].

OS and inflammation have been confirmed in patients with ALD, in several studies. Qu *et al.* [134], observed that heat shock protein 70 (HSP70), an intracellular polypeptide involved in immune responses, contributing to vascular damage and TNF-α release, was increased in alcoholic fatty liver disease. Grasselli *et al.* (2014) [135] also identified a redox imbalance in ALD subjects: increase of lipid peroxidation and stimulation of the activities of the antioxidant enzymes CAT and SOD [135]. Parthasarathy *et al.* (2015) studying cirrhotic patients observed that even during alcohol withdrawal, those alterations are kept [136].

Based on the close relationship between OS and ALD and its progression, research is being conducted to investigate the advantages of antioxidant therapy in animal models as well as in humans. According to Pivetta *et al.* (2006) (Table 2), NAC alone in dose of 300 mg/kg/day, for 30 days, via i.p., restored nonproteic thiols and GPx, which had been reduced with alcohol administration [137]. Another important observation by Caro *et al.* (2014) (Table 2), was the beneficial effects of NAC on OS in cytosol but not in hepatocyte mitochondria [138]. NAC supplementation may be effective during ethanol abstinence improving serum lipids and hepatic antioxidant defenses (GSH/GSSG tissue levels and GR tissue activity) [119] (Table 2). Interestingly, NAC pre-treatment is able to attenuate the lipid peroxidation and GSH depletion caused by the administration of alcohol, in acute period but, in the post-treatment, NAC aggravated hepatic lipid peroxidation and worsened acute ethanol-induced liver damage in a dose-dependent manner [139] (Table 2).

It is clear from the above information that NAC supplementation needs more investigation in hepatic tissue and on several clinical conditions to validate or not its use in ALD, in clinical practice.

**Table 2:** Antioxidant and anti-inflammatory effects of *N*-acetylcysteine in hepatic damage.

Type of Damage	Type of Study	Admin. Route	Dose; Time of Admin	RONS Synthesis or Damage	AO Defense	Cytokines and Interleukins Synthesis and Levels	Ref.
**DISEASES**							
**NASH**	*In vivo* (rats)	Gavage	2 g/kg/d, 65 d	↓ LOOH and MDA tissue levels; ↓ Cytochrome P450 2E1 tissue expression	↑ GSH tissue levels	↓ TNF-α tissue, mRNA expression IL-1β ^#^ mRNA expression	[54]
	*In vivo* (rats)	Oral (diet)	20 mg/kg/d, 6 wk	MDA ^#^ tissue levels	↓ GSH plasm levels	-	[140]
	*In vivo* (rats)	Oral (diet)	500 mg/kg/d, 4 wk	MDA ^#^ tissue levels	GSH ^#^ tissue levels	-	[141]
**Fibrosis**	*In vivo* (rats)	i.p.	50 mg/kg/d, 6 wk	-	GSH ^#^ tissue levels	↓ TNF-α and IL-6 tissue levels	[142]
	*In vivo* (rats)	i.m.	50 μmol/kg/d, 2 wk	↓ TBARS tissue levels; ↓ protein carbonyl tissue levels	↑ GSH and P-SH tissue levels	-	[143]
**Cirrhosis**	*In vivo* (rats)	i.p.	1 g/kg	-	↑ GSH tissue levels	-	[144]
**Diabetes mellitus**	*In vivo* (rats)	i.p.	1.5 g/kg/d, 4 wk after induction	-	↑ TAC plasm levels ↑ SOD tissue activity	↓ TNF-α and IL-6 serum levels	[145]
	*In vivo* (rats)	i.p.	25 ^a^ mg/kg/d; 75 ^b^ mg/kg/d, 30 d	MDA ^#^ tissue levels	↑ GSH tissue levels; GPx ^#^ and SOD ^#^ tissue activities	-	[16]
**Obesity**	*In vivo* (rats)	Oral (water)	2 mg/L/d, 30 d	↓ lipid hydroperoxide tissue levels	↑ Antioxidant capacity; ↑ GSH tissue levels; ↑ GSH/GSSG tissue levels; ↑ SOD tissue activity; CAT ^#^ tissue activity; ↑ GPx tissue activity	-	[146]
**Ischemia-reperfusion**	*In vivo* (mice)	i.p.	300 mg/kg/d, 2 h before ischemia	↓ MDA tissue levels	↑ GSH tissue levels	-	[147]
	*In vivo* (rats)	i.p.	1 g/kg/d, every second day over a 10 day period	↓ Protein carbonyl tissue levels; ↓ Protein carbonyl/GSH tissue levels	↑ GSH tissue levels	-	[148]
	*In vivo* (rats)	i.p.	500 mg/kg, 20 min before induction	↓ MDA tissue levels	↓ GPx tissue activity	-	[149]
	*In vivo* (rats)	i.p.	150 mg/kg, 15 min before ischemia	↓ MDA tissue levels; ↓ protein oxidation tissue; ↓ MPO tissue activity	↑ GSH tissue levels	-	[150]
	*In vivo* (mice)	i.v.	150 mg/kg, 6 ^a^ h, 12 ^b^ h and 24 ^c^ h after IR	↓ NF-κB ^b^ ↓ ROS ^a,b,c^	-	↓ IL-6 ^a,b,c^ mRNA, TNF-α ^a,b^	[151]
**Hepatocellular carcinogenesis**	*In vivo* (rats)	i.p.	100 mg/kg/d, 3 months before induction	↓ ROS	-	-	[152]
	*In vivo* (mice)	Oral (water)	4 mg/mL, starting from 2 months of age	↓ 4-HNE, MDA and 8-OXO-dG tissue levels	-	-	[153]
**Cholestasis**	*In vivo* (rats)	Oral (0.5% carboxymethyl cellulose)	300 mg/kg/d 28 d after bile duct ligation	↓ MDA tissue levels	↑ GSH tissue levels; CAT ^#^ tissue activity	↓ TGF-β and IL-6 tissue expression; IL-10 ^#^ tissue expression	[154]
**Obstructive jaundice**	*In vivo* (rats)	s.c.	100 mg/kg/d, 5 d after induction	↓ MDA tissue levels; ↓ iNOS tissue expression	-	-	[78]
**Acute hepatic failure**	*In vivo* (rats)	s.c.	20 mg/kg, 3 and 6 h after CCl_4_	TBARS ^#^ tissue levels; ↓ protein carbonyls tissue levels	-	-	[155]
	*In vivo* (mice)	i.p.	1.2 g/kg ^a^, immediately before induction and 1.2 g/kg ^b^ injected 1 h after induction	-	↑ GSH ^a^ tissue levels	↓ IL-5 ^a^, IL-10 ^a^, IL-12 ^a^, IL-17 ^a^ and IFN-γ ^a^ tissue levels	[156]
**Schistosomiasis**	*In vivo* (mice)	Oral (water)	300 mg/kg, 5 d a wk/4 wk	-	↓ GSH tissue levels; GST ^#^, GR ^#^, GPx ^#^ and SOD ^#^ tissue activities	-	[157]
**Lipopolysaccharide (LPS)**	*In vivo* (rats)	i.v	150 mg/kg/h (0.3 mL/h) at 60 min and 12.5 mg/kg/h throughout the experiment (0.3 mL/h)	-	-	↓ TNF-α, IL-6 and IL-10 plasma levels	[158]
**ALCOHOL**							
**Ethanol**	*In vivo* (mice)	i.p.	75 ^a^, 150 ^b^ or 300 ^c^ mg/kg/d, at 30 min before ethanol; 75 ^d^, 150 ^e^ or 300 ^f^ mg/kg/d, at 4 h after ethanol	↓ TBARS ^a,b,c^ tissue levels	↑ GSH ^a,b,c^ tissue levels	↓ TNF-α ^a,b,c^ tissue mRNA expression	[139]
	*In vivo* (rats)	Gavage	1.2 g/kg/d, 45 d	↓ MDA and HNE tissue adducts; CYP2E1 ^#^ expression	ORAC ^#^; GSH ^#^ tissue levels	↓ TNF-α tissue mRNA expression	[159]
	*In vivo* (rats)	Gavage	1.2 g/kg/d, for 130 d	-	-	IL-1β ^#^, IL-2 ^#^, IL-4 ^#^, IL-6 ^#^, TNF-α ^#^ levels	[160]
	*In vivo* (rats)	Oral (ethanol solution)	2 g/L with ethanol, 15 d after 30 d of ethanol ^a^, and 2 g/L without ethanol for 15 d after 30 d of ethanol ^b^	-	↑ GSH/GSSG ^a,b^ tissue levels; ↑ GR ^a,b^ tissue activity	-	[119]
	*In vivo* (mice)	i.p.	300 mg/kg/d, 30 d	-	↑ P-SH tissue levels; ↑ GPx tissue activity	-	[137]
	*In vivo* (rats)	Gavage	1.7 g/kg/d, 150 d	Protein carbonyls ^#^ mitochondrial levels; TBARS ^#^ mitochondrial levels; mtDNA ^#^ damage	↑ GSH tissue levels	-	[138]
**DRUGS**							
**Acetaminophen (APAP)**	*In vitro* (hepatocyte)	-	20 mM (dissolved in 10X PBS, pH 7.4) 1 ^a^ h before or 2 ^b^ h after APAP administration	-	↑ GSH ^a,b^ levels	-	[161]
	*In vivo* (rats)	i.p.	100 mg/kg/d, 5 d	-	-	↓ TNF-α and IL-6 tissue levels	[162]
	*In vivo* (rats)	i.p.	2.4 mM/kg/(2 mL dose), 30 min before induction	↓ MDA tissue and serum levels	↑ GSH, GSSG, GSH/GSSG tissue and serum levels; ↑ GR, GST tissue and serum activities	-	[163]
	*In vivo* (mice)	i.p.	1.25 mmol/kg after 1 h after induction	MDA ^#^, ↓ 4-HNE and protein carbonyl tissue levels	↑ GSH and GSSG tissue levels	-	[164]
	*In vitro* (hepatocyte)	-	2.0 mM, 30 min after incubation with APAP	-	↑ GSH levels, P-SH levels	-	[165]
	*In vitro* (hepatocyte)	-	5.0 mM, after 24 ^a^ and 48 ^b^ h of APAP exposure	-	↑ GSH ^a^ levels	-	[166]
	*In vivo* (mice)	i.p.	400 mg/kg, 2 h after induction	-	↑ GSH tissue levels	-	[167]
	*In vitro* (human hepatocyte)	-	250 μM, before 12 ^a^ or 24 ^b^ h	↓ TBARS ^a,b^ levels; ↓ ROS ^a,b^ levels	GSH ^a^ and GSH/GSSG ^#,a^; ↑ GSH/GSSG ^b^ ↑ GR ^a,b^ activity	-	[168]
**Rifampicin**	*In vivo* (rats)	i.p.	100 mg/kg/d, 3 wk	MDA ^#^ levels; Cytochrome P450 2E1 ^#^ tissue levels	SOD ^#^ and CAT ^#^ tissue activities; GPx ^#^ activity GST ^#^ activity GR ^#^ activity	-	[169]
**Azathioprine**	*In vivo* (rats)	i.p.	100 mg/kg/d, 7 d before induction	↓ MDA tissue levels	↑ GSH tissue levels	-	[170]
**Cocaine**	*In vitro* (hepatocyte)	-	0.5 mM, 24 h before incubation with cocaine and 24 h after incubation	↓ Peroxide levels	↑ GSH levels ↑ CAT and GPx RNAm levels	-	[84]
***N*-methyl-methyldopamine**	*In vitro* (hepatocyte)	-	0.1 ^a^ and 1 ^b^ mM, 15 min before induction	-	GSH ^#^ levels	-	[171]
**Cyclosporine A**	*In vivo* (rats)	i.m.	150 mg/kg/d, 11 d starting 1 d before induction	↓ MDA tissue levels; ↓ NO· tissue levels	↑ SOD tissue activity	-	[172]
**Isoniazid**	*In vivo* (rats)	i.p.	100 mg/kg/d, 3 wk	CYP2E1 ^#^ levels	SOD ^#^ and CAT ^#^ tissue activities; P-SH ^#^ levels; GPx ^#^ and GR ^#^ tissue activities	-	[169]
**Statins**	*In vitro* (rats)	-	200 μM	↓ TBARS levels	-	-	[173]
**Methotrexate**	*In vivo* (rats)	i.p.	50 mg/kg/d, 7 d	MDA ^#^ tissue levels	↑ SOD tissue activity; GSH ^#^ tissue levels; CAT ^#^ tissue activity; TAC ^#^	-	[174]
**Carbamazepine**	*In vivo* (rats)	Gavage	50 ^a^, 100 ^b^ and 200 ^c^ mg/kg/d for 45 d	↓ TBARS ^c^ tissue levels	↑ SOD ^c^ and CAT ^c^ tissue activities; ↑ GSH ^c^ tissue levels	-	[175]
**PESTICIDES**							
**Malathion**	*In vivo* (rats)	Oral (water)	2 g/L, 28 d	↓ MDA tissue levels; ↓ MPO tissue activity	↑ GSH tissue levels; ↑ SOD, CAT, GPx and GST tissue activities	↓ IL-1β, IL-6, INF-γ, mRNA levels	[176]
	*In vitro* (hepatocyte)		200 μM, 30 min before exposure	↓ ROS	-	-	[177]
**Paraquat**	*In vivo* (rats)	i.p.	200 mg/kg 2 h before induction	↓ MDA tissue levels; ↓ iNOS tissue levels; ↓ NO^2−^ tissue levels	↑ GSH tissue levels ↓ SOD tissue levels ↑ GR tissue levels ↓ GPx tissue levels	↓ CYP2E1 tissue levels; ↓ TNF-α tissue levels ↓ IL-1β tissue levels	[178]
**Dichlorodiphenyltrichloroethane (DDT)**	*In vitro* (hepatocyte)	-	100 mg/mL, 1 h before of exposure	ROS ^#^	-	-	[179]
**Carbosulfan**	*In vivo* (rats)	Oral (water)	2 g/L for 30 d	↓ MDA tissue levels	↑ GSH tissue levels	↓ IFN-γ mRNA expression	[180]
**IONIZING RADIATION**							
**X-Rays**	*In vivo* (mice)	i.p.	50 ^a^ mg/kg/d, 100 ^b^ mg/kg/d or 200 ^c^ mg/kg/d, 1 h before or after exposure to X-ray irradiation	↓ MDA ^a,b,c^ tissue levels; ↓ DNA ^a,b,c,^* tissue damage	↑ GSH ^a,b,c^ tissue levels 1h before; ↑ GSH ^b,c^ tissue levels after exposure; ↑ SOD ^a,b,c,^* tissue activity	-	[181]
**γ-Rays**	*In vivo* (rats)	i.p	1 g/kg/d, 7 d before exposure to γ-ray irradiation	↓ MDA tissue levels; ↓ DNA tissue damage; (NO(x)) ^#^	↑ GSH tissue levels; ↑ GSH-Px tissue activity; ↑ SOD tissue activity	-	[182]
**OTHERS**							
**Mercury toxicity**	*In vivo* (rats)	i.p.	0.6 g/kg/d, 3 d after induction	↓ MDA tissue levels	↑ SOD and CAT tissue activities; ↑ GSH tissue levels	-	[183]
**Carbon tetrachloride**	*In vivo* (rats)	Gavage	150 mg/kg/d, for 3 months	↓ TBARS plasm and tissue levels; ↓ HP plasm and tissue levels	↑ SOD and CAT tissue activities; ↑ GPx tissue activity; ↑ GSH plasm levels; ↑ Vitamin C and vitamin E plasm levels	-	[184]
	*In vivo* (rats)	i.p.	25 ^a^ mg/kg/d and 50 ^b^ mg/kg/d, 12 wk	↓ LP ^a,b^ tissue levels	↑ GPx ^a,b^ tissue activity ↑ GSH ^a,b^ tissue activity ↑ CAT ^a,b^ tissue activity	↓ CYP2E1 ^a,b^ tissue activity	[185]
	*In vivo* (rats)	i.p.	50 ^a^; 100 ^b^; 200 ^c^ mg/kg/d, 4 wk	↓ MDA ^b,c^ tissue levels	↑ SOD ^a,b,c^ tissue activity; ↑ GSH ^a,b,c^ tissue levels		[186]
**Cadmium (Cd)**	*In vitro* (rats)	-	5 mM, 2 h pre-, simultaneous or 2 h post-treatment	-	↑ GR levels ↑ CAT levels GPx ^#^ levels	-	[187]
	*In vitro* (rats)	-	1 mM ^a^ and 2 ^b^ mM, 1.5 or 24 h, added simultaneously with the Cd	↓ ROS	-	-	[188]
**Glycochenodeoxycholic acid (GCDCA)**	*In vitro* (rats) HepG2 cells	-	0.5 mM, co-administered with GCDCA	↓ O_2_·^−^ production; ↓ NO levels	-	-	[189]
**Methanol**	*In vivo* (rats)	i.p.	150 mg/kg, two doses, after 12 h and 24 h	↓ MDA tissue levels	↑ GSH tissue levels ↑ GSSG tissue levels ↑ GPx tissue activity	-	[190]
**Fluoride**	*In vitro* (rats) culture	-	1 mmol/L, 60 ^a^ min before and 60 ^b^ min simultaneously with fluoride	↓ MDA ^a^ tissue levels	↑ GPx ^a^ tissue levels ↑ GR ^a^ tissue levels	-	[191]
**iNOS**	*In vivo* (rats)	s.c.	100 mg/kg, 5 d	↓ MDA tissue levels; ↓ iNOS tissue expression	-	-	[78]
**Dimethylnitrosamine**	*In vivo* (rats)	Gavage	50 mg/kg/d, 7 d	↓ MDA tissue levels	↑ SOD tissue activity; ↑ Vitamin C tissue levels; ↑ Vitamin E tissue levels; ↑ GPx tissue activity; ↑ GSH tissue levels; ↑ CAT tissue activity; ↑ GST tissue activity	-	[192]
**Arsenic**	*In vivo* (rats)	i.p.	10 mg/kg/d, for 3 wk	TBARS tissue levels ^#^	CAT tissue activity ^#^ GSH tissue levels ^#^	-	[193]
**Mercuric chloride**	*In vitro* (mice)	-	10 ^a^–500 ^b^ μM	↓ MDA ^b^ levels	-	-	[194]
**Polychlorinated biphenyls**	*In vivo* (rats)	Oral (diet)	1% (10g/kg diet) one wk before induction	CYP1A1 ^#^ activity	↓ GSSG/GSH; GST ^#^ tissue activity	-	[145]
**Isoflurane anaesthesia**	Human	i.v.	12.5 mg/kg/h throughout the operation (laparoscopic surgery)	↑ MDA plasm levels	↑ GST plasm levels; ↑ GSH plasm levels	-	[195]
**Cardiopulmonary bypass**	*In vivo* (rats)	-	250 mg/kg, at 0.5, 1, 2, 3, and 24 h	↓ MDA tissue levels ↓ MPO tissue levels ↓ iNOS tissue levels	↑ GSSG tissue levels ↑ GSH tissue levels ↑ GPx tissue activity	-	[196]
**Acrylamide (ACR)**	*In vivo* (rats)	Gavage	250 mg/kg/d, 21 d	↓ MDA tissue levels	↑ GSH tissue levels; ↑ GST tissue activity		[197]

^a^,^b^,^c^,^d^,^e^ = different treatments; * = group that presented more beneficial action; - = not available; (−): decrease; ^#^ = there wasn’t significant difference when compared to the positive control; ↑ = increased; ↓ = decreased; b.w.: body weight; CP4502E1: cytochrome P450, family 2, subfamily E, polypeptide 1; CAT: catalase; d: days; GR: glutathione reductase; GPx: glutathione peroxidase; GSH: reduced glutathione; GSH/GSSG: ratio reduced glutathione/oxidized glutathione; GST: glutathione *S*-transferase; h: hours; HNE: Hydroxynonenal; HP: hydroperoxide; i.v.: intravascular; i.p.: Intraperitoneal; i.m.: intramuscular; iNOS: inducible nitric oxide synthase; IL: interleukin; INF-γ:Interferon gamma; kg = kilogram; LP: lipid peroxide; MPO: Myeloperoxidase; mRNA: messenger RNA; mtDNA: Mitochondrial DNA; MDA: malondialdehyde; MnSOD: manganese superoxide dismutase; min: minute; NASH: non-alcoholic steatohepatitis; (NO(x)): total nitrate/nitrite; NF-κB: factor nuclear κ B; ng = nanogram; NO: nitric oxide; ORAC: the oxygen radical absorbance capacity; P-SH: Protein Sulfhydryl; protCo: protein carbonyls; PBS: phosphate buffered saline; Ref.: reference; ROS: reactive oxygen species; SOD: superoxide dismutase; s.c.: subcutaneously; TAC: antioxidant capacity; TNF-α: tumor necrosis factor α; TBARS: thiobarbituric acid-reactive substances; TGFβ1: transforming growth factor β 1; *vs.*: *versus* ; 8-OHdG: 8-hydroxy-2′-deoxyguanosine; 4-HNE: 4-hydroxy-2-nonenal; wk: week.

#### 4.3.3. Non-Alcoholic Steatohepatitis

Nonalcoholic fatty liver disease (NAFLD) is one of the most common chronic liver diseases worldwide [198]. NAFLD refers to a clinic pathologic spectrum of conditions ranging from simple steatosis (fatty liver) to non-alcoholic steatohepatitis (NASH), involving inflammation, liver cell damage, and in some cases, cirrhosis and HCC [199]. It is estimated that NAFLD affects 20%–30% in the general population, and 70%–90% in obese or diabetic patients [200]. The pathogenesis of NAFLD, especially NASH, is multifactorial and includes lipid metabolism alterations, insulin resistance (IR), increase of inflammation, OS and production of advanced glycation end products (AGEs) [9,33,201].

The role of inflammation and OS in pathogenesis of NAFLD may be confirmed by the “two-hit” theory by Day *et al.* (1998) [202]. According to this theory, and cited by Santos *et al.* (2015) [9], the first hit concerns triglycerides (TG) and free fatty acids (FFA) accumulation into hepatocytes, caused by the increase of IR, enhanced dietary influx and increased hepatic lipogenesis; whereas the second hit is related to lipid peroxidation, mitochondrial dysfunction and inflammation, which result in hepatocyte damage and development of liver fibrosis.

RONS have the function of activating and inhibiting signaling pathways that can modulate the metabolism of lipids, especially the progression of steatohepatitis, caused by the increase of adipokines and immune system activation [33]. Another important point is that RONS may induce signaling pathways that may trigger IR [203].

Patients with NAFLD/NASH present several alterations in redox and inflammatory markers, such as increase of high sensitivity C-reactive protein (hsPCR) [204]; AGE [205], which may result from a variety of reactions (direct way) or by mechanism involving the hydroxy radical-mediated oxidation of lipids (indirect way) [206] and MDA [207]. Besides that, the decrease of TAS [208] and SOD was also observed [209].

It is possible that NASH can occur in the absence of simple steatosis because inflammation related to NASH pathogenesis might originate in gut microbiota, in response to the infiltration of neutrophil chemokines and macrophage-inflammatory protein-2, inflamed adipose tissue and to circulating inflammatory cells [210]. One mechanism of hepatic inflammation may result in ROS via cytokine driven inflammation with predominance of ROS generation by CYP2E1 [211]. The enzyme CYP2E1 has its expression increased in NASH, and this significantly increase the levels of ROS [9].

As mentioned above, hepatocytes are equipped with multiple defense systems, allowing a protection against oxidative processes [212]. In this review, 3 papers about NAC and NAFLD/NASH and all in animal models were found.

Samuhasaneeto *et al.* (2001) [141] (Table 2) observed that rats in a high fat diet (HFD) model of NASH induction and that received NAC (500 mg/kg/day), orally, had the levels of total GSH and hepatic MDA back to normal levels. However, the addition of NAC was not better than diet treatment alone (diet with normal fat) [141]. In another animal model, inducing NASH by HFD via enteral nutrition, Baumgardner *et al.* (2008) (Table 2), using NAC (2 g/kg/day) demonstrated that this antioxidant had prevented many aspects of NASH progression by decreasing development of OS (decreased MDA) but it was unable to block development of steatosis [54]. Thong-Ngam *et al.* (2007) (Table 2), in a diet-induced NASH (using a 100% fat diet), concluded that GSH in NASH + NAC (20 mg/kg/day, for 6 weeks) group was significantly lower than in the NASH group [140].

The liver is more exposed to hyperinsulinemia than any other tissue because insulin is transported via the portal vein [213]. Steatosis and NASH are present in 95% and 20% of obese patients, respectively [42].

Diniz *et al.* (2006) [146] (Table 2) examined whether sucrose-rich diet (SRD) induced hyperglycaemia, dyslipidemia and OS in rats. It was observed that the oral administration of 2 mg of NAC/L/day in water, for 30 days, decreased lipid hydroperoxide (LOOH) hepatic levels and increased the antioxidant capacity of liver, through the increase of the GSH hepatic levels and SOD and GPx hepatic activity, thus increasing the reductive capacity and inhibiting LOOH alteration in hepatic tissue in rats. NAC, due to its antioxidant activity, inhibits the metabolic shifting and induces beneficial effects on obesity. According to this study, the administration of NAC in food may be feasible and beneficial. Additional studies are required to understand the mechanisms of action of NAC in obesity, aiming its prescription as a medicine to attenuating the harmful effects of obesity.

Type 1 (T1DM) and type 2 diabetes (T2DM) are associated with increased risk of chronic liver injury, including the NAFLD [214]. NAC was tested in rat model of streptozotocin-induced T1DM, since GSH decrease may have an important role in the development of diabetic complications, occurring in early diabetes. The administration of 1.5 g of NAC/kg/day, via gavage, for 4 weeks after induction of diabetes, increased TAS levels, in plasm, and SOD hepatic tissue activity, and inflammatory biomarkers levels were decreased in serum, for instance, TNF-α and IL-6. SOD activity and protein expression in diabetes is dependent on the tissue. Inflammation is a key process of the progression of diabetes. Some inflammatory factors, such as TNF-α and IL-6, are elevated in patients with T2DM, and correlate with the incidence of diabetic macrovascular complications. Thus, NAC may confer protection against OS by restoring or increasing SOD enzyme activity. NAC also presented anti-inflammatory property [145] (Table 2). However, Ribeiro *et al.* (2011) (Table 2) evaluating the effects of 25 mg of NAC/kg/day and 75 mg of NAC/kg/day, for 30 days, administered i.p., in decreasing oxidative tissue damage in the liver of alloxan-induced diabetic rats, observed that there was no effect on MDA levels and GPx and SOD tissue activities, although it has increased GSH tissue levels [16].

All these results together stimulate new research about NAC and NAFLD in animal models and in humans, once this process is closely connected with NAFLD pathogenesis and morbidity.

#### 4.3.4. Lipopolysaccharides

LPS is a prototypical pathogen-associated molecular patterns (PAMPs), which is present in the outer membrane of Gram-negative bacteria [35,44], and has been reported to exert inflammatory responses, fibrosis, and hepatocellular damage [215]. In conditions, such as an increase in intestinal permeability associated with dysbiosis [216], and ulcerative colitis [217], LPS transport from the gut to target tissues, also known as bacterial translocation [44]. As liver is an organ closely linked to gut through portal vein, it becomes the direct target of an intestinal inflammation, resulting in the induction and progression of liver injury [215,218].

It is suggested that endotoxemia is responsible for the initiation of hepatic damage through the interaction of endotoxin with Toll-like receptors (TLR) [218,219]. TLR comprise a family of pattern recognition receptors (PRPs), which recognize bacterial, viral and fungal components. Of these, TLR4 has a central role in the activation of Kupffer cells, by responding to LPS [38].

An important effect of recognition of LPS by TLR4 is the activation of NF-κB, resulting in the production of pro-inflammatory cytokines [220], such as TNF-α, which in turn leads to the development of hepatic steatosis [219,221], by modulating sterol regulatory element-binding proteins, (SREBP), being the SREBP-1c the main form expressed in the liver. These proteins are a family of membrane-bound transcription factors, which activate genes encoding enzymes involved in lipid synthesis [222].

In the study by Hsu *et al.* (2006) [158], to induce an endotoxin shock in Wistar–Kyoto rats, through the administration of 10 mg of LPS/kg by a slow intravenous infusion, the post-treatment with NAC, by an i.v, drip at a rate of 150 mg/kg/h (0.3 mL/h), at 60 min and 12.5 mg/kg/h throughout the experiment (0.3 mL/h), significantly decreased plasma levels TNF-α and IL-6, after LPS administration, and NAC significantly increased the plasma IL-10, an anti-inflammatory cytokine, showing that NAC modulates the events signaling of LPS. As a consequence, post-treatment with NAC may exert beneficial actions against LPS-induced organ damage, as the liver, by reducing the levels of pro-inflammatory cytokines and increasing anti-inflammatory cytokines. However, studies are still needed to evaluate the antioxidant and anti-inflammatory role of NAC in liver damage caused by bacterial translocation, such as the imbalance of the intestinal microbiota (intestinal dysbiosis) and ulcerative colitis, to understand the mechanism of action of NAC on liver in endotoxemia and so to establish a safe therapy.

#### 4.3.5. Intoxication

##### Drug-Induced Liver Injury

The hepatotoxic effect of some therapeutic drugs is widely recognized as a health problem [32,223]. Most cases of drug-induced liver injury (DILI) do not occur in a predictable dose-dependent manner, leading to a delayed recognition of hepatotoxicity induced by drug [173,224]. DILI may also resemble the complete set of acute or chronic liver diseases, such as hepatitis and cholestasis [225]. The incidence of DILI in general populations is around 14–19/100,000 inhabitants [226,227]. The drug properties and host factors have an important role in DILI development [225,228].

In the case of drugs’ properties, the factors that lead to lesions include surpassing a threshold dose, physicochemical characteristics, reactive metabolites formation, OS, depletion of antioxidants and interference on mitochondrial respiration [223,229]. The OS generated in DILI may be due to the cytosolic stress during drug metabolism and/or subsequent response by injured liver cells [223]. The best example of DILI is represented by acetaminophen (APAP) [32,229]. The OS generated by this drug is recognized to generate *N*-acetyl-*p*-benzoquinone imine (NAPQI) by cytochrome P450, one toxic metabolite that shows oxidative capacity through attack and covalent modification of proteins [32], as well as reduction of GSH/GSSG ratio by oxidizing the thiol group of GSH [163]. Moreover, APAP cause hepatotoxicity through the generation of ROS and RNS, and peroxidation reaction products [163].

Drug toxicity can also induce an inflammatory response through the production of cytokines by KCs and neutrophils, such as TNF-α, IL-6, IL-1β, IL-1α, and IFN-γ that modulate intracellular events [162]. These cytokines sensitize hepatocytes to biochemical stress, or regulate the adaptive immune-mediated cell injury. Moreover, drug-protein adducts are presented as an antigen, triggering the adaptive immune response by binding to T-cell receptors of CD4 cells, leading to CD8 cytotoxic T-cell activation [225].

NAC is an antidote against hepatotoxicity caused by APAP overdose, in clinical practice. There are many studies reporting the roles of NAC in APAP-induced liver injury. According to the study of Taslipinar *et al.* (2013) [162] (Table 2) that evaluated the hepatoprotective effects of NAC in an overdose of APAP producing centrilobular hepatocellular necrosis in rats, it was observed that doses of 100 mg/kg/day, i.p. for 5 days, significantly reduced hepatic TNF-α and IL-6. These cytokines were released in APAP-induced liver injury and were responsible for damage in liver. Based on these results, NAC has shown hepatoprotective effects. In the work of Acharya *et al.* (2010) [162] (Table 2), NAC increased hepatic and serum levels of GSH and GSH/GSSG ratio, and liver and serum activities of GR, GST, and decreased hepatic and serum levels of MDA. NAPQI is capable of decrease GSH/GSSG ratio through the oxidation of the thiol group of GSH, and can also lead to the formation of interstrand disulfide linkages, in protein cross-links and protein-GSH mixed disulfides by oxidizing Cys thiol groups in proteins. APAP-associated diminution of the intrahepatic GSH is accompanied by reduction in the activities of the antioxidant enzymes GR, CAT, GPx and SOD γ-glutamylcysteinyl synthetase (GCS). NAC administered as a single dose, 0.783 mg/kg/(2 mL), 30 min before administration of APAP, in this study, promoted protective actions against the toxic actions of APAP in the liver (Table 2).

The immunosuppressive agent cyclosporine A (CsA) was reported to exert measurable hepatotoxic effects, by stimulation of ROS formation and depletion of hepatic antioxidant defense. In the work of Kaya *et al.* (2008) [172] (Table 2) the effects of NAC treatment on CsA-induced hepatic damage in rats in dose of 150 mg/kg/day, i.m., for 11 days, starting 1 day before induction, were investigated. A decrease of NO· and MDA hepatic levels, as well as an increase of SOD liver activity were observed. The membrane lipid peroxidation, a mechanism of cell death, was found to be increased by CsA. NAC treatment protected against the toxicity caused by CsA administration, through its antioxidant and radical scavenging action.

##### Pesticides

Many pesticides are toxic to mammals once they are not fully selective to the target organism. Chronic exposure to pesticides can lead to various pathological conditions including liver damage [178,230]. Organophosphate pesticides (OPS) are the most commonly used insecticides in the world, and generally are the most toxic of all pesticides to vertebrates. Pesticides can impact human health through many ways such as residual proximity to agricultural pesticide application, accumulation in food production and water supply [231].

OPS, such as malathion, induce hepatotoxicity, OS and liver inflammation with enhancement of liver expression of pro-inflammatory cytokines. Liver structure and function are affected by the pathological lesions caused by malathion leading to hepatosteatosis. It induces a high ROS production in the liver when metabolized to malaoxon through oxidative sulfuration. Necrotic changes resulting in hepatomegaly have been reported. Rise in liver weight has also been reported possibly due to the intensity of damage or collagen accumulation. All these findings are related to OS [176]. Toxicity to isolated hepatocytes with enhancement of MDA production, and depletion of GSH activity were induced by trichlorfon and dichlorvos (both OPS) administration [232]. OPS also demonstrate genotoxic and alkylating properties in liver. They can induce apoptosis through decrease of mitochondrial energy, proteolytic enzyme induction and DNA fragmentation [233,234].

There are few studies in literature evaluating the effect of NAC against hepatic damage caused by pesticides through OS. However, in these cases, NAC was shown to have attenuated the level of hepatotoxicity markers (including LP and nitrite content) induced by paraquat (a widely used bipyridyl herbicide) and maneb (a dithiocarbamate fungicide) via OS [178] (Table 2). In addition, NAC restored liver enzyme activity and attenuated OS markers in malathion-induced liver damage [176] (Table 2) and prevented ROS formation in the cells treated with 1 mM malathion [177] (Table 2).

These data are promising, however more studies are needed to enable the application of NAC in the treatment of liver damage induced by pesticides.

##### Toxins and Miscellaneous Chemicals

The contact with toxins or chemicals such as carbon tetrachloride (CCl_4_) or metals in humans is not so common and normally happens in the industry during production and in places where those chemicals are often used [10].

Most metals released into the atmosphere by industries are toxic and bioaccumulative. As examples, heavy metals such as cadmium (Cd), arsenic (As) and mercury (Hg), which are disseminated in the environment, since the beginning of the Industrial Revolution until the current days, lead to tissue damage by species generated through reactions with these metals. The type and aggravation of this damage will depend on the amount and exposure time to these metals [183].

Results of the work of Joshi *et al.* (2014) [183] (Table 2) showed that NAC protects against Hg toxicity evaluated by reducing MDA hepatic tissue levels and increase of SOD and CAT liver tissue activities and GSH liver tissue levels, preventing oxidative degradation of biological membranes. NAC protects the cells from induced toxicity by Cd, by sequestration of free radicals [188] (Table 2) or by increasing the antioxidant enzyme levels, as can be seen in some works [187] (Table 2). Studies *in vivo* are necessary to evaluate the antioxidant role of NAC and so, along with clinical trials, generate more safety in its recommendation as a protective drug against Cd-induced damage in liver.

CCl_4_ is activated by cytochrome CYP2E1 to form the trichloromethyl radical (CCl_3_·) and reacts with various macromolecules interfering negatively in cellular metabolism such as the lipid one, leading to hepatic steatosis [235]. NAC had shown a protective effect in CCl_4_-induced hepatotoxicity in rats, through the reduction of TBARS in plasm and tissue levels and increase of the antioxidant defenses SOD, CAT and GPx tissue activities and GSH, vitamin C and vitamin E plasm levels that were found decreased upon CCl_4_ administration [170]. Depletion the levels of vitamin C and vitamin E may indicate increased OS and generation of ROS in CCl_4_-induced liver injury. Similar results were found by Nissar *et al.* (2013) [185] (Table 2) where, in addition to increasing the antioxidant defenses, they evaluated the anti-inflammatory effect of NAC, demonstrating that it caused a decrease in CYP2E1 hepatic tissue activity. In case of chronic intoxication by CCl_4,_ the hepatic levels of CYP2E1 are significantly increased. The increase of OS results from the formation of reactive metabolites due to the biotransformation by CYP2E1. ROS/RNS lead to lipid peroxidation, triggering a cascade of reactions intensifying the OS. NAC treatment showed a significant decrease in LP levels, showing an effect of NAC on CCl_4_ intoxication.

#### 4.3.6. Ischemia/Reperfusion

Ischemia-reperfusion (I/R) injury, one condition characterized by disruption of blood in an organ with consequent absence of nutrient and oxygen supply [236], leads to liver damage. This process occurs in some situations, such as during surgery, like in hepatic resection and liver transplantation, and in some diseases, such as ischemic hepatitis and multiple organ failure [237]. When oxygenation and blood flow are restored, the damage caused during the period of ischemia intensifies, worsening the injury produced at the cellular level [238,239]. I/R is associated with increased OS and mitochondrial dysfunction [8]. ROS are generated initially during reperfusion and lead to serious damage to tissues. Endoplasmic reticulum (ER) stress mediates liver damage during liver I/R injury and ROS can trigger ER stress *in vivo* and *in vitro* [147]. In addition, ROS produced during I/R can stimulate apoptosis, the main cause of cell death, associated with a caspase-dependent pathway [240].

The last step of I/R injury is initiated by neutrophil-mediated proteases, TNF-α, and IL-1β. Moreover, there are microcirculatory disorders, formation of xanthine oxidase, stimulation of NADPH oxidase by activated KCs and neutrophils [8,241]. I/R causes liver damage by induction of RNS. The NO formation by iNOS may cause peroxynitrite-induced damage and also lead to cell injury through direct inhibition of mitochondrial respiratory chain enzymes [236,242]. Ischemia stimulates KCs, that are the central cells responsible for ROS release during the reperfusion period [236].

Sun *et al.* (2014) [147] (Table 2) evaluated the effects of NAC on ER stress and tissue injury during liver I/R injury. Using a dose of 300 mg NAC/kg/day, 2 h before ischemia in mice via i.p., a decrease of MDA and an increase of GSH levels in hepatic tissue, were observed.

Additionally, Fernández *et al.* (2013) [148] (Table 2) noted that the administration of 1 g NAC/kg/day, via i.p., every second day over 10 day period in rats, attenuated protein carbonylation in hepatic tissues and increased GSH hepatic tissue levels. In addition, Sener *et al.* (2003) [150] (Table 2) evaluated a dose of 150 mg NAC/kg, for 15 min before ischemia via i.p., in *Wistar* rats and observed that NAC caused the decrease of MDA hepatic tissue levels, protein oxidation tissue and hepatic myeloperoxidase (MPO) activity which were raised by I/R, and the increase of GSH tissue levels.

These findings show that NAC plays a protective role in the liver injured by I/R and can be a possible therapeutic agent in hepatic I/R.

#### 4.3.7. Hepatocelullar Carcinoma

Hepatocellular carcinoma (HCC) is the fifth most common cancer in the world. It is caused by a malignant transformation of hepatocytes, the major cell type in the liver, and accounts for 80% to 90% of primary liver cancer [243]. HCC may occur in patients with HBV [127], alcohol [244], NASH [211] and mainly HCV [245]. Many steps associated with chronic inflammation are involved in the pathogenesis of hepatocarcinomas [128]. Among these processes, OS stands out, contributing to the advance of the clinical state of disease through telomere shortening in hepatocytes and enhancing the malignant features of HCC, such as high proliferative activity and apoptotic resistance by telomerase activation in cancer cells [246].

Therefore, OS may play an important role in some intracellular signaling cascades such as oxidation of DNA with generation of modified DNA molecules like 8-oxo-7,8-dihydro-2-deoxyguanosine (8-OXOdG) that has a mutagenic effect in mammalian cells, and can therefore be considered carcinogenic [247]. OS also have a close action on cell signaling, especially with growth factors, cytokines such as TNF-α and IL-1β, transcription factors like NF-κB and AP-1, and regulates matrix metalloprotease 1 (MMP1), a protease that has the ability to destroy and invade connective tissue. All of them increased OS with generation of RONS and/or they are stimulated by OS, and consequently increase apoptosis and finally, cause carcinogenesis [248]. Besides OS, inflammation had been shown in studies of animal models of chemically-induced HCC.

Lin *et al.*, studying HCC in toll-like receptor 2 (TLR2) deficient mice, observed that ROS/ERstress is directly responsible for the worsening of liver carcinogenesis, and NAC significantly attenuated these effects (Table 2). Another important discovery of these authors was that NAC treatment interrupts the positive feedback of the ROS/ER stress-p62 aggregation-unfolded protein response (UPR)-induced inflammation and backed the TLR2 deficiency increased susceptibility of HCC development and progression. It is known that UPR (unfold protein response) imbalance is directly involved in ER stress. The authors concluded that OS in ER is closely involved in the development of HCC and NAC is able to prevent it [248] (Table 2).

This antioxidant has not been studied in subjects with HCC or to prevent it, but has been tested in other cancer such as head and neck [249], colon [250]. However, the results are not conclusive. Combined to this, the use of antioxidants during cancer treatment is still controversial in the scientific community [251]. All results together suggest that more studies about NAC chemoprevention in HCC are required, first in different HCC induced-models and after in humans.

## 5. Concluding Remarks

Based on the above discussion and in results listed in Table 2, several remarks can be written, which might lead to some rationalization or design of further studies, challenges and perspectives in the use of NAC in OS-based diseases.

### 5.1. Type of Study

The *in vivo* models (rats and mice) were widely used in experimental studies due to their prolificacy, easy management and maintenance, known biology and genome, being less expensive. These qualities, along with the similarity of the results to humans, support the use of these species as good animal models for experimentation. However, it is important to have a deep understanding of the biology of the species, because, in this way, you can design the best goals and the most appropriate experimental protocol, making these animals models, experimentally reliable.

Despite the limitation on the number of experimental variables, *in vitro* studies are simpler and faster than *in vivo* testing and can serve as a preliminary study. Therefore, they are widely used in toxicologic studies, and, in this review, *in vitro* studies have investigated NAC action on the damage caused by toxins, pesticides and drugs. In these cases, the use of NAC has showed very favorable results in the reduction of the levels of markers related to ROS generation and on the damage and increase defense in the liver. However, these studies should also take into consideration, markers of inflammation, since they are related to OS. These results suggest that NAC is a good antidote to such poisoning and radiation in animal models (Table 2).

Although *in vitro* experiments indicate relevant antioxidant abilities for NAC, it is questionable if the methods underestimate physiological antioxidant capacity. Before a systematic use, several studies are still necessary to evaluate the bioactivity of NAC at hepatic injury, mostly on animals and human beings.

### 5.2. Doses, Route of Administration and Study Time

**Figure 5:**
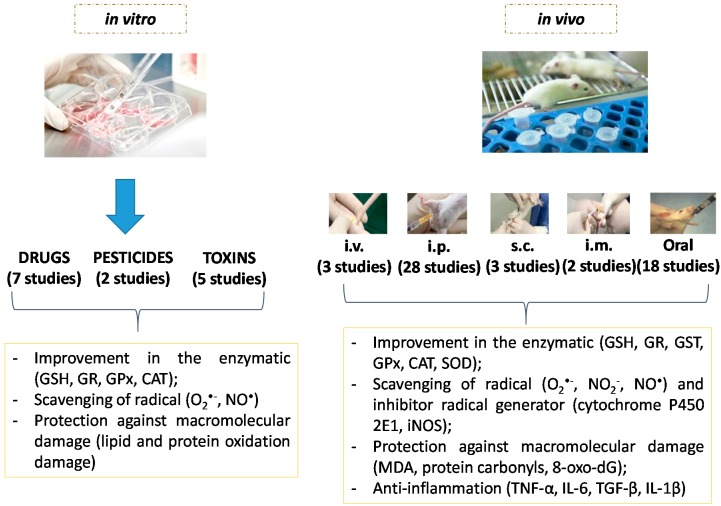
Positive effects of *N*-acetylcysteine *in vivo* and *in vitro* tests evaluated in this review and its mains results.

Use of NAC is attractive due to its wide availability, ease of administration, and low cost. A variety of doses (from around 0.204 g/kg/day up to 2 g/kg/day total doses at *in vivo* studies) and routes of administration have also been used (Table 2). The intraperitoneal route was the most used in these studies (Figure 5). The supply of substances in animal to studies is frequently a serious component of experimental design. Therefore, some information is mandatory for administration of substances in animals. This is a general procedure. ADMET parameters (absorption, distribution, metabolism and excretion of the substance), together with route, volume, frequency of administration, duration of treatment, pH, stability, homogeneity, and osmolality of the substance to be administered, selection of vehicle or solvent for substance delivery (in case it cannot be administered in a solid or particulate state), solution preparation [252], among others, need to be considered. The absorption, distribution and elimination of i.p. supplied compounds are more similar to those observed after oral ingestion, because the main route of absorption is into the mesenteric vessels, which drain into the portal vein and pass through the liver [253]. Therefore, the compounds that are provided by i.p. may go through hepatic metabolism before their arrival in the systemic circulation [254].

In the study by Wang *et al.* [139] it was observed that NAC had an enhancing effect on the hepatic injury in the post-treatment. Further studies are required to confirm this result.

Due to the broad variation in terms of dose, route and length of administration of NAC, evidenced by analysis of Table 2, it would be clarifying if each study informs the reason for any decision regarding those parameters.

This requires the standardization of the model study, according to each type of hepatic injury, and to evaluate the optimal dose for the treatment of chronic and acute cases, allowing greater security in the selection of dose.

### 5.3. Biomarkers

Table 1 lists the most common used biomarkers. Although a compelling body of evidence indicates that OS is involved in pathways leading to hepatic damage, there is no agreement among scientists in terms of the best and most accurate measure of OS. One common attitude used in evaluating OS is to measure the decreases of endogenous antioxidants. Indeed the supplementation with NAC is effective for increasing GSH levels [255], confirmed in this review, independent on the type and time of the study and route of administration. As such, this antioxidant is the most indicated as a marker of OS. The GSH/GSSG can be also used as an *in vitro* and *in vivo* indicator of the redox balance in cells and, consequently, of cellular OS [256].

To properly assess inflammation, the markers should be effective. They should reproduce the inflammatory process in study and be predictive of future health status. Many of the processes, cells and molecules involved in the inflammatory response caused by different agents leading to liver damage, mentioned in this review, are remarkably similar. They are characterized by an increase in the number of leucocytes in the bloodstream and hepatic tissue and by overproduction and presence in the bloodstream of increased levels of inflammatory cytokines (TNF-α, IL-1β, IL-6 and IFN-γ) and chemokines (IL-8 and MCP-1) [257]. The increase of the levels of these mediators acts to amplify the inflammatory process and contribute to tissue destruction. These markers are common to inflammation, independent of cause.

## 6. Challenges and Perspectives

Liver diseases are highly prevalent in the world, and the beneficial results of studies both *in vivo* and in clinics in this direction could strongly benefit patients, considering that NAC is a safe, cheap and well tolerated antioxidant, with a well-defined mechanism of action. The controversy about beneficial roles in some diseases and not in others should be clarified. To allow comparison, in terms of treatment and protective effects, reliable positive and negative controls might be chosen.

Few clinical studies on the antioxidant and inflammatory potential of NAC in humans have been carried out (Table 2). Additionally, the absence of studies that evaluated the attenuation of oxidative stress markers and inflammation by NAC in hepatitis B and C, and in bacterial translocation, and few studies in obesity and diabetes, raises difficulties for prescription of NAC to patients. Before that, studies are necessary to ensure. Therefore, further studies are required to explore the use of NAC, using the most recent comprehensive analysis of genomic and transcriptomic techniques, which will allow further investigation on how NAC affects metabolic pathways.

Due to the good results found in this review for NAC as attenuator of markers of oxidative stress, especially on GSH levels, and inflammation, additional efforts both *in vivo* and in humans, in chronic diseases, are required to allow assessment of NAC as a potential therapeutic drug, in liver injury. As a challenge, for new studies, a validated design for the treatment and in protection models should be discussed and implemented, for instance, the presence of a control group. It should also be considered the route of administration, which provides more beneficial effects in individuals affected by these conditions. The increase in clinical studies and evaluation of oxidative stress and inflammation markers in the serum are urgently required. Such findings will provide helpful information on the health benefits of NAC on liver injury to furnish data to professionals in terms of effectiveness and safety of the drug to reach a safe and reliable future prescription.

## Abbreviations

ADH: alcohol dehydrogenase; ACR: acrylamide; AGEs: advanced glycation end products; ALD: alcoholic liver disease; ALDH: aldehyde dehydrogenase; ALT: alanine aminotransferase; AP-1: activator protein 1; APAP: acetaminophen; AO: antioxidants; αSMA: smooth muscle α-actin; AUC: concentration-time curve; b.w.: body weight; CsA: cyclosporine A; CAT: catalase; Cit c: cytochrome c; CKD: chronic kidney disease; CLDs: chronic liver diseases; CP4502E1: cytochrome P450, family 2, subfamily E, polypeptide 1; COPD: chronic obstructive pulmonary disease; COX: cyclooxygenase; Cp: core protein,; CsA: cyclosporine A; Cys: cysteine; Cys–Cys: cysteine; d: day; De-Acase: deacetylases; DCV: cardiovascular diseases; DILI: drug-induced liver injury; DNA: deoxyribonucleic acid; DDT: Dichlorodiphenyltrichloroethane; ECM: extracellular matrix; ER: endoplasmic reticulum stress; FFA: free fatty acids; GCDCA: Glycochenodeoxycholic acid; GCS: γ-glutamyl cysteinyl synthetase; GPx: glutathione peroxidase; GR: GSH reductase; GST: glutathione S-transferase; GSH: reduced glutathione; GSSG: oxidized glutathione; GSH/GSSG: ratio reduced glutathione/oxidized glutathione; HBV: hepatitis B virus; HCC: hepatocellular carcinoma; HCV: hepatitis C virus; HIV: human immunodeficiency virus; HFD: high fat diet; HP: hydroperoxide; HBx: X protein; HSCs: hepatic stellate cells; hsPCR: high sensitivity C-reactive protein; Hsp90: protein 90; IkB-α: nuclear factor of kappa light polypeptide gene enhancer in B-cells inhibitor α; IKK: IκB kinases; IKKβ: inhibitor of κB kinase; IκB: inhibitor of NF-κB; IL: interleukin; IL-1R: interleukin-1 receptor; i.m.: intramuscular; i.p.: intraperitoneal; I/R: ischemia-reperfusion; IR: insulin resistance; INFγ: interferon gamma; iNOS = inducible nitric oxide synthase; i.v.: intravascular; JNKs: c-jun N-terminal kinases; KCs: Kupffer cells; LPO: lipoxygenase; LP: lipid peroxide; LPS: lipopolysaccharide; LOOH: hydroperoxide; MCP-1: monocyte chemoattractant protein 1; MDA = malondialdehyde;MMP1: matrix metalloprotease 1; MPO: myeloperoxidase; NADPH oxidase: nicotinamide adenine dinucleotide phosphate-oxidase; NADP^+^: oxidized nicotinamide adenine dinucleotide phosphate; NAC: *N*-acetyl-l-cysteine; NF-κB = nuclear factor κ-light-chain enhancer of activated B cells; NAFLD: nonalcoholic fatty liver disease; NASH: non-alcoholic steatohepatitis; NIK: NF-κB-inducing kinase; NK: natural killer; NKT: natural killer T cells; NAPQI: N-acetyl-p-benzoquinone imine; NP-SH: non-protein sulphadryls; NO(x): Total nitrate/nitrite; Nrf2: Nuclear factor (erythroid-derived 2)-like 2; OPS: Organophosphate pesticides; OS: oxidative stress; OSI: oxidative stress index; ORF: open reading frames; PDGF: platelet-derived growth factor; PMN: polymorphonuclear leukocytes; P-SH: thiol protein; PUFAs: polyunsaturated fatty acids; p65: nuclear factor NF-κB p65 subunit; PGE2: prostaglandin; p50: nuclear factor NF-κB p50 subunit; PON1: paraoxonase-1; Pol: polymerase; RHD: domain homology; PAMPs: pathogen-associated molecular patterns; PRPs: pattern recognition receptors; P-SH: thiolated protein; ROMs: reactive oxygen metabolites; RONS = reactive oxygen and nitrogen species; RNS: reactive nitrogen species; ROS: Reactive oxygen species; R-SH: thiol; Rel A: v-rel avian reticuloendotheliosis viral oncogene homolog A; Ref.: reference; ROOH: organic hydroperoxide; ROH: alcohol; SREBP: sterol regulatory element-binding proteins; SOD: superoxide dismutase; STAT-3: signal transducer and activator of transcription 3; s.c.: subcutaneous; Sp: surface protein; SRD: sucrose-rich diet; TNF-α: tumor necrosis factor α; TLR2: toll-like receptor 2; TLR4: toll-like receptor 4; T2DM: type 2 diabetes; T1DM: type 1 diabetes; TNF-R1: TNF-α receptor 1; TGF-β: transforming growth factor β; TG: triglycerides; TIMP1: tissue inhibitor of metalloproteinase-1; TAS: total antioxidant status; TOS: total oxidant status; TAC: antioxidant capacity; T-GSH: total glutathione sulphydryls; UPR: unfolded protein response; VEGF: Vascular endothelial growth factor; VCAM-1: vascular cell adhesion molecule 1; XO: xanthine oxidase; w.k.: Week; 4-HNE: 4-hydroxy-2-nonenal; 8-OHdG: 8-oxo-7,8-dihydro-2-deoxyguanosine; CO_3_·^−^: carbonate radical; HO· = hydroxyl radical; HOCl: hypochlorous acid; H_2_O_2_ = hydrogen peroxide; O_2_: singlet oxygen; O_2_·^−^ = superoxide anion; ONOO^−^: peroxynitrite; ONOOH: peroxynitrous acid; NO· = nitric oxide; NO_2_·: nitrogen dioxide; ΔΨ: mitochondrial membrane potential; 1: NADH-ubiquinone reductase; 2: succinate-ubiquinone reductase; 3: ubiquinol-cytochrome c reductase; 4: cytochrome c oxidase.
